# The role of frontal and parietal cortex in the performance of gifted and average adolescents in a mental rotation task

**DOI:** 10.1371/journal.pone.0232660

**Published:** 2020-05-13

**Authors:** Renata Figueiredo Anomal, Daniel Soares Brandão, Silvia Beltrame Porto, Sóstenes Silva de Oliveira, Rafaela Faustino Lacerda de Souza, José de Santana Fiel, Bruno Duarte Gomes, Izabel Augusta Hazin Pires, Antonio Pereira

**Affiliations:** 1 Brain Institute, Federal University of Rio Grande do Norte, Natal (RN), Brazil; 2 Department of Psychology, Federal University of Rio Grande do Norte, Natal (RN), Brazil; 3 Maurício de Nassau College, Natal (RN), Brazil; 4 Department of Electrical and Biomedical Engineering, Institute of Technology, Federal University of Pará, Belém (PA), Brazil; 5 Institute of Biology, Federal University of Pará, Belém (PA), BRAZIL; Universita degli Studi di Roma La Sapienza, ITALY

## Abstract

Visual-spatial abilities are usually neglected in academic settings, even though several studies have shown that their predictive power in science, technology, engineering, and mathematics domains exceeds that of math and verbal ability. This neglect means that many spatially talented youths are not identified and nurtured, at a great cost to society. In the present work, we aim to identify behavioral and electrophysiological markers associated with visual spatial-ability in intellectually gifted adolescents (N = 15) compared to age-matched controls (N = 15). The participants performed a classic three-dimensional mental rotation task developed by Shepard and Metzler (1971) [33] while event-related potentials were measured in both frontal and parietal regions of interest. While response time was similar in the two groups, gifted subjects performed the test with greater accuracy. There was no indication of interhemispheric asymmetry of ERPs over parietal regions in both groups, although interhemispheric differences were observed in the frontal lobes. Moreover, intelligence quotient and working memory measures predicted variance in ERP’s amplitude in the right parietal and frontal hemispheres. We conclude that while gifted adolescents do not display a different pattern of electroencephalographic activity over the parietal cortex while performing the mental rotation task, their performance is correlated with the amplitude of ERPs in the frontal cortex during the execution of this task.

## I. Introduction

Spatial thinking is “the ability to generate, retain, retrieve, and transform well-structured visual images.” [1]. Reliance on visuospatial thinking is high among science, technology, engineering, and mathematics (STEM) professionals [2]. For instance, Albert Einstein famously used visual “thought experiments” to formulate his theory of relativity and continued to use them throughout his remarkable career. A longitudinal study showed that high levels of spatial ability in youths predict long-term interest in STEM [3, 4] and the strong association between spatial thinking and competence in mathematics and associated disciplines [5] led to the proposal that spatial learning be integrated into school curricula [5] and used in talent searching for adolescents with potential for STEM [4]. Even though spatial ability is a strong predictor of success in STEM [4, 6], quantitative and verbal reasoning abilities are still favored over non-verbal abilities in traditional educational settings [7], even when searching for gifted and talented students [4, 8].

There is ample consensus that the gifted and talented are relevant contributors to a dynamic, knowledge-based society and that they should be given adequate support in both academic and familial settings. Nowadays, many countries harbor institutions to promote initiatives associated with the identification and education of gifted children and youths [9]. However, many challenges remain for gifted education, especially in less developed regions of the world [10]. Traditionally, most definitions of giftedness refer to the construct of general intelligence and, as a result, gifted individuals have been characterized through a narrow operational definition, such as scoring above 130 on standardized intelligence tests [11]. There is widespread understanding, however, that giftedness should not be measured by a single intelligence quotient (IQ) [12] but should be understood within a human-developing framework [9, 13, 14], contingent on the availability of nurturing environments [12– 15].

Intelligence can be defined as the ability to reason, plan, solve problems, think abstractly, comprehend complex ideas, learn quickly and learn from experience [16]. The Cattell–Horn–Carroll (CHC) Theory of Human Cognitive Abilities [17] proposes a hierarchical structure with three levels or strata encompassing major (broad ability) and minor (narrow ability) sources of individual intelligence differences [17, 18]. At the top of the hierarchy lies general intelligence, also known as *g* [19]. The middle stratum is composed of abilities that are more specific than *g*, including visual processing, or *Gv*, associated with the use of visualization, or mental imagery, to solve problems [20]. A study by Gustafsson (2001) [21] showed that students who had completed technical and science tracks in secondary school had higher *Gv* scores than students from non-vocational tracks, emphasizing the close association of Gv with STEM.

Understanding the neural basis of human intelligence is one of the main challenges of modern Neuroscience. Early studies had already associated intelligence with both functional and structural aspects of individual frontal [22] and parietal [23] brain regions. Other more recent studies have focused on the interaction between these regions organized in functional networks [24], such as the frontoparietal network [24–27]. Not surprisingly, the activity of the frontoparietal network has been associated with visuospatial abilities and mental imagery during the performance of event-related tasks [28–32].

After its introduction by Shepard and Metzler (1971) [33], mental rotation using abstract geometric figures is one the most successful experimental paradigms in cognitive neuroscience and its performance measures are strongly affected by mathematical ability [34–36]. It is proposed that performance on mental rotation tasks reflect the functionality of underlying neural processes involved with imagery rather than the individual’s previous knowledge of the situation [37]. A study with functional magnetic resonance imaging (fMRI) suggested that, during mental rotation, mathematically gifted adolescents engage the frontoparietal network differently from youths with average math ability [30]. Later works confirmed these findings, associating individual differences in mathematical abilities with a distinct pattern of activation of the frontoparietal-network [38–42].

Differences in both the amplitude and latency of event-related potentials (ERP) can be used to make inferences about the timing and nature of cortical stimulus processing under different experimental conditions [43, 44]. Mental rotation reliably activates regions in the parietal cortex [45] and the amplitude of ERP decreases over parietal scalp locations as a function of stimulus orientation [46]. According to Heil (2002), the onset of rotation-related negativity around 400 ms post-stimulus onset [48, 49] can be used as a chronopsychophysiological marker for mental rotation [47]. However, as shown by Milivojevic and colleagues (2009), the mental rotation interval may vary in latency and duration in accordance with the experimental design and should be calculated for each experiment [50].

The goal of the present study is twofold: first to establish the degree of association between visuospatial ability and intelligence measures in adolescents. Second, to describe the electrophysiological signature in the frontoparietal network associated with visuospatial ability. We used a mental rotation task based on the Shepard-Metzler paradigm as a proxy for visuospatial ability, while also measuring ERPs in both frontal and parietal regions of interest (ROIs). We hope this work can help bring to light brain phenotypes associated with intellectual giftedness and use it for the identification of gifted youths. Our results can also provide new insights into the structures and systems associated with aspects of human intelligence [51].

## II. Methods

### 2.1 Participants

All procedures were approved by the Ethics Committee of the Federal University of Rio Grande do Norte (UFRN) (CAAE: 50197415.9.0000.5537). All participants gave their informed written consent for participation in the study. For minors, consent was signed by either their parents or legal guardians. The participants had no neurological dysfunction and had no uncorrected visual impairments.

Participants were assigned to the experimental groups according to scores on Wechsler intelligence scales (WISC and WAIS). The total IQ score is composed of the following index subscores: (1) verbal comprehension, (2) perceptual organization, (3) working memory and (4) processing speed [52, 53].

Total IQ scores equal and above 130 were considered “very superior”, between 120 and 129 “superior”, and between 80 and 119 “average” [54]. The volunteers recruited for the gifted group (N = 15) were adolescents (13–21 y.o.) with total IQ equal or above 129. These participants were enrolled in the Gifted Program of the Digital Metropolis Institute of UFRN. The control group (N = 15) was age-matched with the gifted group and had total IQ scores between 80 and 128.

### 2.2 Experimental design

**Fig 1:**
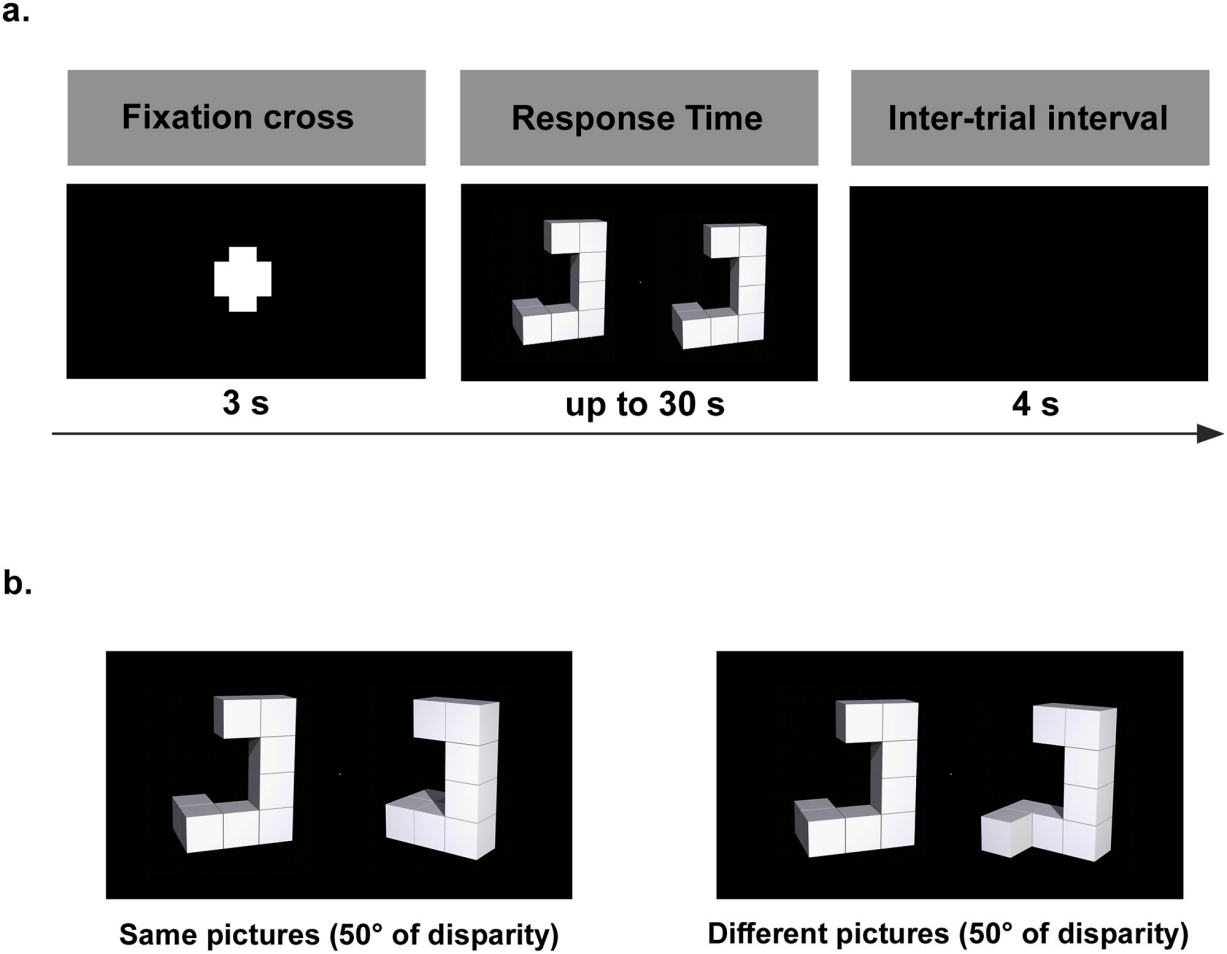
(A) Experimental design. (B) Experimental conditions: “same” and “different” stimulus pairs (B). Based on [55].

Participants performed a classic Shepard-Metzler mental rotation task [33], while having their electroencephalogram (EEG) simultaneously recorded (see below). The stimuli were presented on an LCD computer monitor (1920x1080 pixels) located 0.90 to 1.00 m in front of the participant. The experiment contained 160 trials organized randomly. Fig 1 illustrates the sequence of events per trial: (1) appearance of a fixation cross for 3 seconds, (2) display of stimulus for up to 30 seconds, and (3) inter-trial interval of 4 seconds [55]. When response times took more than 30 seconds, the current trial was aborted and a new one was initiated by the software (Psychopy, v1.90.d).

Each trial was composed of two pictures presented side-by-side (see Fig 1B). The pictures were similar to the ones used by Ganis and Kievit (2015) [56]. Participants were instructed to judge if the pictures were “same” or “different”. In both the “same” or “different” experimental conditions, the picture on the right side was rotated relative to the left picture and could be either normal or mirror reversed. Pictures were rotated clockwise around the longitudinal axis in 50° increments from 0° to 150° (angle disparity). For both conditions, a total of 80 trials were presented, with 20 trials for each angle disparity (0°, 50°, 100°, and 150°).

Prior to the task, participants were briefed about the experiment’s design with a slide presentation and were instructed: 1) to perform a mental rotation to solve the test, 2) to press either the right or the left button of the mouse for the “same” or “different” condition, respectively, 3) to respond as fast as possible, and 3) to avoid committing errors.

### 2.3 Electroencephalographic recording

The EEG was recorded continuously during the task with a 1000 Hz sampling rate from 64 Ag/AgCl electrodes mounted on a cap according to the international 10–20 system (BrainAmp system, Brain Products). The electrode EOGz was placed at the glabella, and the electrodes EOG1 and EOG2 were positioned lateral to the left and right eye, respectively. The EEG recordings occurred in a darkened room, with sound attenuation and temperature control. The electrical impedance was kept under 25k Ohms for all electrodes. During acquisition, the signals were referenced to the electrode FCz. EEG data were analyzed with the EEGLab toolbox running in Matlab version 7 (Mathworks, Inc.). Channels were re-referenced to the mean of all electrodes and band-pass filtered between 0.1–35 Hz. Ocular and muscular artifacts were removed through Independent Component Analysis (ICA) (S1 Fig). Electrodes with consistently poor signal quality were removed and reconstructed with interpolation using the PREP pipeline tool of the EEGLab toolbox.

The ERP signal was epoched to 200 ms before stimulus onset and 4000 ms post-stimulus onset. For baseline correction, we subtracted the average potential amplitude of the 200 ms interval immediately preceding the stimulus onset from each epoch. Data from error trials and those with a signal amplitude above 100 μV were excluded from the analysis. We included in the analysis only participants who performed at least 120 trials (75% of total trials).

### 2.4 Data analysis

Individual performance during the mental rotation task was represented by both accuracy and response time (RT) measures. Accuracy was the percentage of the participant’s correct responses during the experimental session. Group accuracy was calculated by averaging individual participants’ results. Accuracy values for group, picture condition, and angle disparity were analyzed with a three-way mixed analysis of variance (ANOVA), using the group as between-subject and picture condition or angle disparity as within-subject factors. RT represents the average time per session taken by each participant from the stimulus presentation until the mouse button was pressed. Trials with incorrect responses were not considered for calculating RT. The RT of groups was calculated by averaging the individual participants’ results. The RT for picture condition or for angle disparity was analyzed by three-way mixed ANOVA, using groups as between-subject and picture condition or angle disparity as within-subject factors.

We used stepwise multiple linear regression to identify whether IQ score (total IQ) and its subscores (working memory, perceptual organization, processing speed, and verbal comprehension) predicted accuracy and/or RT values obtained during the visuospatial task. We performed a correlation analyses between behavioral measures (accuracy and RT for “same” pictures) and cognitive test scores (total IQ, working memory, perceptual organization, processing speed and verbal comprehension) from pooled participants’ data. We used Cook’s distance (Cook’s D) to identify outliers in the multiple linear regression and correlation analyses. Accuracy and RT values with a Cook’s D larger than 0.13 (4/n, considering n total = 30) were removed from analysis.

The time window associated with ERP’s rotation-related negativity was determined as 963–1183 ms for “same” pictures and as 1132–1252 ms for “different” pictures [50]. The time window in which voltage was linearly related to orientation was determined according to the method described by Milivojevic and coworkers (2009) [50] as the interval that exceeded in 20% the negative peak of the grand-averaged linear ERPs. The ERP of individual subjects for each stimulus orientation (0°, 50°, 100° and 150°) was multiplied by linear weight constants -3, -1, 1, 3, respectively, and the values obtained for each orientation were scaled by the square root of the sum of the squares of the weights.

The interhemispheric effects for “same” pictures during rotation-related negativity were obtained by analyzing pairs of homologous electrodes located at opposite hemispheres (pair 1: P1-P2, pair 2: P3-P4, pair 3: P5-P6, pair 4: P7-P8). A two-way mixed ANOVA (2 x 2) was performed using groups as between-subject and hemisphere (by pairs of electrodes) as within-subject factors. We also analyzed group and hemispheric effects between pairs of frontal electrodes (pair 1: F1-F2, pair 2: F3-F4, pair 3: F5-F6, pair 4: F7-F8) during the rotation-related negativity interval of “same” pictures condition using two-way mixed ANOVA (2 x 2).

For parietal (P1 to P8 and Pz) and frontal (F1 to F8 and Fz) electrodes, effects of group, picture condition and angle disparity were analyzed during the rotation-related negativity intervals. Comparisons were performed with three-way mixed ANOVA (2 x 2 x 4), considering the variable group as between-subject and picture condition and angle of disparity (0°, 50°, 100°, and 150°) as a within-subject factor.

Stepwise multiple linear regression was used to verify whether total IQ and its subscores were predictor of ERPs amplitude of parietal (P1, P2, P3, P4, P5, P6, P7, P8, Pz) and frontal electrodes (F1, F2, F3, F4, F5, F6, F7, F8, Fz). Correlation analyses between these electrodes and intelligence scores were also done for the “same” picture condition. Both gifted and control groups were considered together for all correlation analyses. Cook’s D was calculated to remove any data with large influence on the multiple linear regression or correlation analyses (Cook’s D > 0.13).

For the analyses of scalp topography, we performed a two-way repeated measures ANOVA with the variable group as between-subject and electrodes as within-subject factors. The electrodes used for the electro-oculogram were excluded from topographic analyses. Topography was analyzed using standard and normalized data.

### 2.5 Statistical analysis

We used the Shapiro-Wilk normality test to evaluate whether the data followed a normal (Gaussian) distribution. We calculated the mean squared error (MSE) and effect size (partial eta-squared: partial η2) for the ANOVAs. We used the Sidak’s Method as a multiple comparison post-hoc test. The Bonferroni post-hoc correction was used for multiple comparisons with significance cut off at 0.05/n. For the correlation analysis, we used the Pearson’s correlation coefficient was used when data followed a normal distribution or its non-parametric alternative, the Spearman’s rank coefficient. Orthogonal polynomial contrasts were used to reveal any of linear, quadratic, or cubic trends for the variables. Data are presented as mean ± SEM (standard error of the mean) and the criterion for significance was set at 0.05.

## III. Results

Female and male participants were analyzed together. Participants were either right or left-handed, with right-handedness being more prevalent in our sample (Table 1). Total IQ for the control group ranged from 94–121 and 129–143 for the gifted group. Average IQ of control and gifted groups are shown in Table 1.

**Table 1:** Summary of characteristics in the participant sample.

Group	Sex (%)		Hand Dominance (%)		Age (y.o.)	I.Q.
	Male	Female	Right	Left		
**Control**	10 (66.67)	5 (33.33)	12 (80)	3 (20)	16.27 ± 0.42	107.6 ± 2.10
**Gifted**	9 (60)	6 (40)	12 (80)	3 (20)	16.20 ± 0.56	136.0 ± 1.44

### 3.1 Behavioral analysis

**Fig 2:**
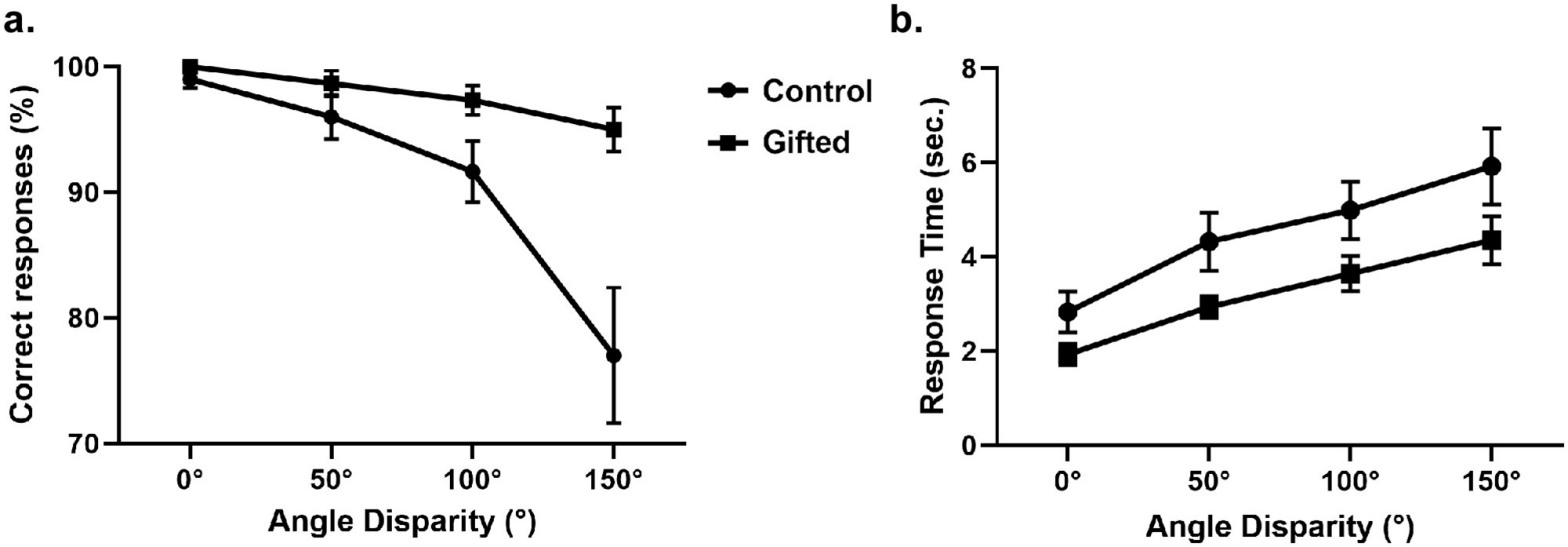
Accuracy and RT comparison between groups. Accuracy (A) and RT (B) for “same” pictures condition by angle disparity.

There was a statistically significant interaction among group, angle disparity and picture condition (F (1, 28) = 7.114, MSE = 137.6558, p = 0.004, partial η2 = 0.203) on accuracy for the “same” picture condition (Fig 2A, Table 2). Conversely, there was no significant interaction between group and picture condition (F (1, 28) = 0.276, MSE = 137.6558, p = 0.604, partial η2 = 0.010), group and angle disparity (F (1, 28) = 2.302, MSE = 137.6558, p = 0.083, partial η2 = 0.076) or picture condition and angle disparity (F (1, 28) = 3.392, MSE = 137.6558, p = 0.054, partial η2 = 0.108). Still on the “same” picture condition, there was a significant effect of angle disparity (F (1, 28) = 19.191, MSE = 137.6558, p < 0.001, partial η2 = 0.407) and group (F (1, 28) = 10.233, MSE = 137.6558, p = 0.003, partial η2 = 0.268), but not of picture condition (F (1, 28) = 3.903, MSE = 137.6558, p = 0.058, partial η2 = 0.122). Regarding accuracy for “same” pictures, the angle disparity effect could be described by linear (F (1, 28) = 22.288, p < 0.001) and quadratic trends (F (1, 28) = 11.715, p = 0.02). The interaction between group and angle disparity also showed a linear (F (1, 28) = 9.187, p = 0.005) and quadratic trend (F (1, 28) = 9.319, p = 0.005). Neither the angle disparity effect (F (1, 28) = 1.437, p = 0.241) nor the interaction between angle disparity and group could be described by a cubic trend (F (1, 28) = 1.068, p = 0.310) (The results of accuracy and RT for “different” pictures condition are shown in S2 Fig and S1 Table).

**Table 2:** Behavioral results for accuracy.

Accuracy (%)					
Group	Total	“Same” pictures			
		0°	50°	100°	150°
**Control**	88.83±1.340	99.00 ±0.724	96.00±1.773	91.67±2.423	77.00±5.407
**Gifted**	96.63±0.513	100.0±0.000	98.67±1.041	97.33±1.182	95.33±1.723

The analysis of RT (Fig 2B, Table 3) showed no significant interaction between group, picture condition and angle disparity (F (1, 28) = 1.762, MSE = 6.612, p = 0.161, partial η2 = 0.059), between group and picture condition (F (1, 28) = 0.629, MSE = 6.612, p = 0.435, partial η2 = 0.022) or between group and angle disparity (F (1, 28) = 0.719, MSE = 6.612, p = 0.543, partial η2 = 0.025). A statistically significant interaction was found between picture condition and angle disparity (F (1, 28) = 16.38, MSE = 6.612, p < 0.0001, partial η2 = 0.369). While no significant group effect was observed in RT (F (1, 28) = 3.532, MSE = 6.612, p = 0.071, partial η2 = 0.112), significant effects were found on picture condition (F (1, 28) = 14.60, MSE = 6.612, p = 0.001, partial η2 = 0.343) and angle disparity (F (1, 28) = 49.20, MSE = 6.612, p < 0.0001, partial η2 = 0.637).

**Table 3:** Behavioral results for RT.

RT (sec)					
Group	Total	“Same” pictures			
		0°	50°	100°	150°
**Control**	5.056±0.282	2.830±0.432	4.324±0.617	4.99±0.610	5.924±0.807
**Gifted**	3.569±0.149	1.932±0.234	2.934±0.233	3.640±0.377	4.354±0.513

As to RT for the “same” picture condition, the angle effect could be described by linear and quadratic trends (F (1, 28) = 80.941, p < 0.001 for linear trend and F (1, 28) = 5.296, p = 0.029 for quadratic trend). The angle disparity effect could not be described by a cubic trend (F (1, 28) = 2.046, p = 0.164). For the interaction between angle disparity and group, a trend analysis showed no significant linear (F (1, 28) = 0.984, p = 0.330), quadratic (F (1, 28) = 0.544, p = 0.467), or cubic trends (F (1, 28) = 0.654, p = 0.426).

We used multiple linear regression to verify whether intelligence scores could explain the variance of accuracy and RT values. During the “same” picture condition, a regression analysis did not result in a statistically significant model for accuracy (F (1, 25) = 3.643, p = 0.068, R^2^ = 0.127) or for RT values (F (1, 25) = 2;303, p = 0.142, R^2^ = 0.084) (The multiple linear regression analysis of behavioral results during “different” pictures condition is shown in S2 Table).

**Fig 3:**
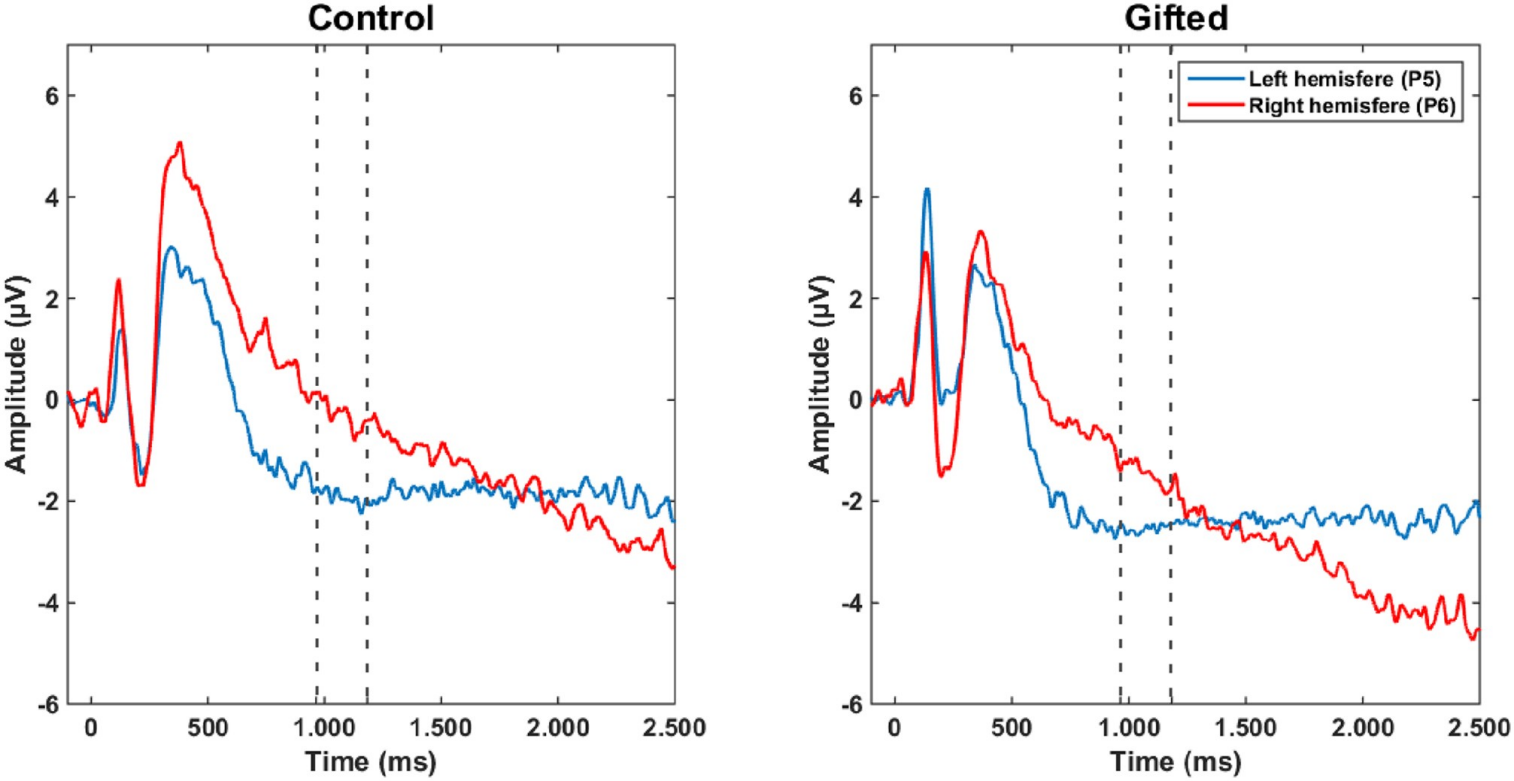
Grand-averaged ERPs over electrodes P5-P6. ERPs recorded in the control (left) and gifted (right) groups over the electrodes P5-P6 during the “same” pictures condition. Values of rotation-related negativity indicate the average ERP amplitude of each group.

We also analyzed the correlation between accuracy and intelligence scores (Fig 3). Our results showed no correlation between accuracy during the “same” picture condition with total IQ (r = + 0.332, p = 0.091, Spearman test), working memory (r = +0.122, p = 0.545, Spearman test), processing speed (r = +0.136, p = 0.499, Spearman test), perceptual organization (r = + 0.336, p = 0.086, Spearman test), and verbal comprehension (r = + 0.269, p = 0.175, Spearman test). In the “same” picture condition, there was no correlation between RT and total IQ (r = -0.345, p = 0.078, Spearman test), working memory (r = -0.112, p = 0.577, Spearman test), processing speed (r = -0.127, p = 0.527, Spearman test) and perceptual organization (r = -0.307, p = 0.119, Spearman test), and verbal comprehension (r = -0.365, p = 0.061, Spearman test).

### 3.2 Event-related potentials

#### 3.2.1 Interhemispheric analysis of ERPs during rotation-related negativity

The analysis of interhemispheric distribution of ERPs over parietal electrodes during the “same” picture condition (Fig 3, Table 4) showed no interaction between group and hemisphere over the electrodes P1-P2 (F (1, 28) = 0.601, MSE = 12.609, p = 0.445, partial η2 = 0.021), P3-P4 (F (1, 28) = 0.043, MSE = 8.595, p = 0.838, partial η2 = 0.002), P5-P6 (F (1, 28) = 0.211, MSE = 9.861, p = 0.650, partial η2 = 0.007), and P7-P8 (F (1, 28) = 1.113, MSE = 12.676, p = 0.300, partial η2 = 0.038). Also, no hemispheric effect was found between P1-P2 (F (1, 28) = 0.176, MSE = 12.609, p = 0.678, partial η2 = 0.006), P3-P4 (F (1, 28) = 0.148, MSE = 8.595, p = 0.703, partial η2 = 0.005), P5-P6 (F (1, 28) = 0.004, MSE = 9.861, p = 0.951, partial η2 < 0.001), and P7-P8 (F (1, 28) = 1.661, MSE = 12.676, p = 0.208, partial η2 = 0.056).

**Table 4:** Interhemispheric comparison of rotation-related negativity for parietal and frontal electrodes during “same” pictures condition.

		Parietal				Frontal	
Electrodes	Group	Hemisphere	Group x Electrodes	Electrodes	Group	Hemisphere	Group x Electrodes
**P1-P2**	0.634	0.678	0.445	**F1-F2**	0.758	0.906	0.960
**P3-P4**	0.490	0.703	0.838	**F3-F4**	0.730	0.013*	0.268
**P5-P6**	0.362	0.951	0.650	**F5-F6**	0.720	0.008*	0.634
**P7-P8**	0.732	0.208	0.300	**F7-F8**	0.996	0004*	0.807

p values,

* statistically significant

In frontal electrodes (Table 4), there was no interaction between group and hemisphere over the electrodes F1-F2 (F (1, 28) = 0.003, MSE = 11.660, p = 0.960, partial η2 < 0.001), F3-F4 (F (1, 28) = 1.278, MSE = 11.660, p = 0.268, partial η2 = 0.044), F5-F6 (F (1, 28) = 0.232, MSE = 19.029, p = 0.634, partial η2 = 0.008), and F7-F8 (F (1, 28) = 0.061, MSE = 17.346, p = 0.807, partial η2 = 0.002). However, hemispheric effects were found over electrodes F3-F4 (F (1, 28) = 7.078, MSE = 19.029, p = 0.013, partial η2 = 0.202), F5-F6 (F (1, 28) = 8.015, MSE = 19.029, p = 0.008, partial η2 = 0.223) and F7-F8 (F (1, 28) = 9.923, MSE = 17.346, p = 0.004, partial η2 = 0.262).

**Fig 4:**
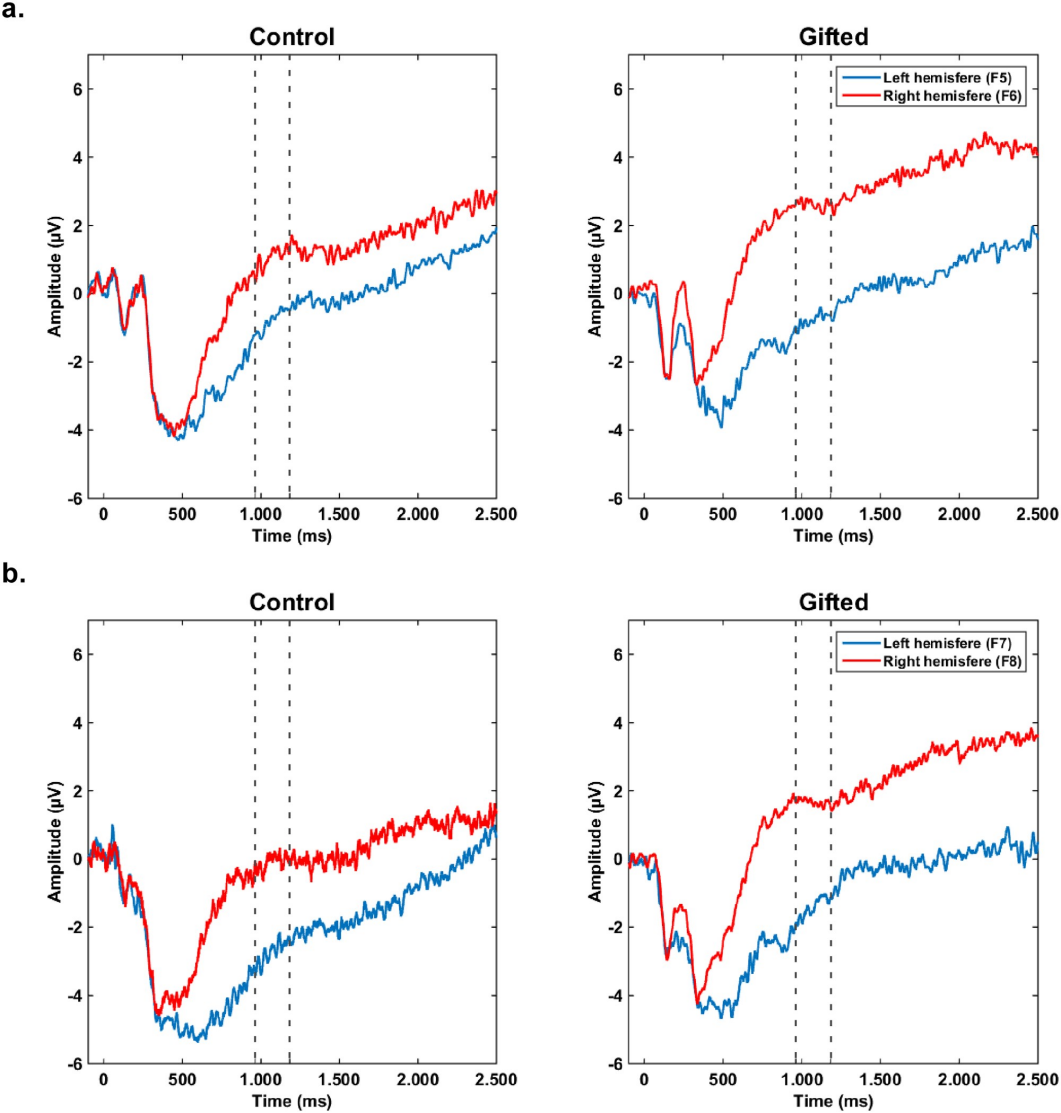
Grand-averaged ERPs over frontal electrodes. ERPs recorded in the control (left) and gifted (right) groups over the electrodes F5-F6 (A) and F7-F8 (B) during the “same” pictures condition. Values of rotation-related negativity indicate the average ERP amplitude of each group.

After post hoc correction, only hemispheric effects over the electrodes F5-F6 and F7-F8 were considered (adjusted critical value for significance p < 0.0125). The ERP amplitude was lower in electrode F5 (-0.271 ± 1.113 μV) than in F6 (1.846 ± 0.558 μV). The ERP amplitude over the electrode F7 (-1.281 ± 0.774 μV) was lower than over F8 (1.195 ± 0.733 μV) (Fig 4).

#### 3.2.2 ERPs during rotation-related negativity

As shown in Table 5, the analyses of ERP amplitudes during rotation-related negativity over the parietal electrodes showed interaction between group and angle disparity.

**Table 5:** Results of parietal analyses during rotation-related negativity interval.

	Group	Angle Disparity	Picture condition	Group x Angle disparity	Group x Picture condition	Picture condition x Angle disparity	Group x Picture condition x Angle disparity
**P1**	0.310	0.549	0.175	0.045*	0.998	0.357	0.637
**P2**	0.968	0.361	0.018*	0.037*	0.873	0.298	0.686
**P3**	0.720	0.496	0.745	0.974	0.191	0.324	0.277
**P4**	0.530	0.757	0.044*	0.055	0.905	0.235	0.585
**P5**	0.666	0.901	0.720	0.255	0.495	0.170	0.148
**P6**	0.384	0.898	0.113	0.761	0.986	0.494	0.698
**P7**	0.812	0.632	0.451	0.747	0.729	0.943	0.121
**P8**	0.456	0.828	0.295	0.409	0.457	0.757	0.361
**Pz**	0.673	0.432	0.005*	0.437	0.236	0.307	0.836

p values after post hoc test,

* statistically significant

**Fig 5:**
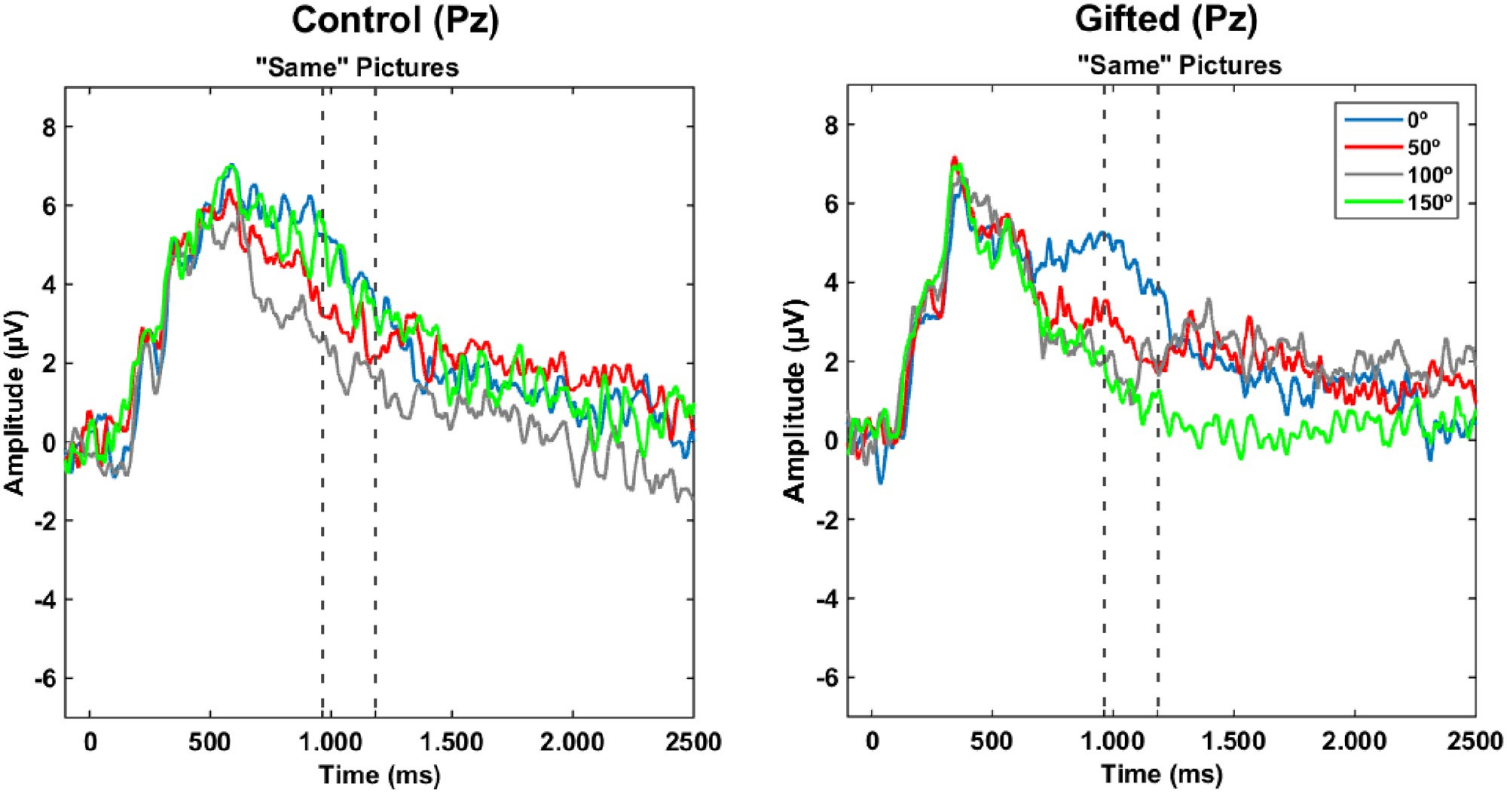
Grand-averaged ERPs separated by picture condition and angle disparity over the electrode Pz. ERPs recorded in electrode Pz during the “same” picture condition for control (A) and gifted adolescents (B). The average ERPs of each angle of rotation (0°, 50°, 100° and 150°) is color-coded. Values of rotation-related negativity indicate the average of the absolute amplitude of ERPs’ for each group.

An interaction between group and angles disparity was found over the electrodes P1 (F (1, 28) = 2.792, MSE = 16.344, p = 0.045, partial η2 = 0.477) and P2 (F (1, 28) = 2.955, MSE = 16.344, p = 0.037, partial η2 = 0.477). Also, there were effects of pictures condition over the electrodes P2 (F (1, 28) = 6.294, MSE = 31.056, p = 0.018, partial η2 = 0.303), P4 (F (1, 28) = 4.450, MSE = 16.344, p = 0.044, partial η2 = 0.477) and Pz (F (1, 28) = 9.520, MSE = 23.760, p = 0.005, partial η2 = 0.285). After post hoc correction, however, and considering the adjusted critical value for significance p < 0.005 (0.05/9), only picture condition effects over the electrode Pz was considered (Fig 5, S3 Fig).

The angle disparity effect for electrode Pz could not be described by any of the linear (F(1,28) = 284, p = 0.598 for the linear trend), quadratic (F(1,28) = 1.922, p = 0.177), or cubic trends (F(1,28) = 0.066, p = 0.799). The interaction between angle disparity and group showed a significant cubic trend (F(1,28) = 0.429, p = 0.518 for linear trend; F(1,28) = 0.027, p = 0.870 for quadratic trend; F(1,28) = 4.254, p = 0.049 for cubic trend).

During the “same” picture condition in frontal electrodes, there was no interaction between group and picture condition or angle disparity (Table 6), between group and angle disparity, group and picture condition or picture condition and angle disparity. Effects of group, picture condition or angle disparity were not found in frontal electrodes.

**Table 6:** Results of frontal analyses during rotation-related negativity.

	Group	Angle Disparity	Picture Condition	Group x Angle disparity	Group x Picture Condition	Picture condition x Angle disparity	Group x Picture condition x Angle disparity
**F1**	0.955	0.301	0.297	0.202	0.465	0.889	0.583
**F2**	0.870	0.660	0.521	0.705	0.118	0.361	0.938
**F3**	0.808	0.935	0.237	0.824	0.349	0.859	0.687
**F4**	0.631	0.867	0.154	0.820	0.058	0.228	0.817
**F5**	0.988	0.746	0.479	0.969	0.795	0.579	0.605
**F6**	0.748	0.590	0.358	0.422	0.372	0.897	0.481
**F7**	0.892	0.393	0.865	0.247	0.748	0.665	0.986
**F8**	0.762	0.760	0.911	0.366	0.638	0.852	0.534
**Fz**	0.775	0.782	0.099	0.900	0.055	0.770	0.915

p values after post hoc test,

* statistically significant

### 3.3 Topography of ERPs

**Fig 6:**
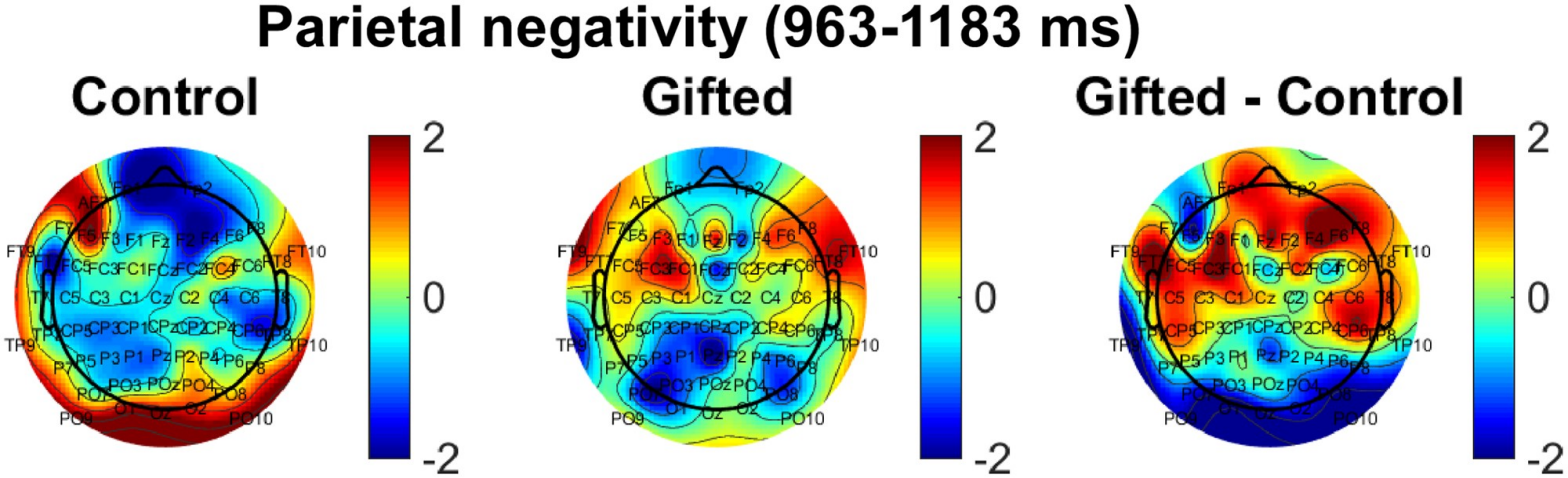
Scalp topographies of ERP amplitudes. EEG scalp topography of rotation-related negativity (963–1183 ms) for the “same” picture condition for controls (left) and gifted subjects (middle). Te difference of ERP amplitude between the gifted and control groups is displayed to the right. Values are color-coded according to normalized ERP amplitude.

During rotation-related negativity intervals for the “same” picture conditon (Fig 6), from 963–1183 ms, the cortical topographical maps for the control group displayed a large spread negative wave spreading over the parietal ROIs, especially over the left hemisphere. In the gifted group, areas of negative potential were observed over the left and right parietal cortex. After subtracting the average ERP amplitude of the gifted and control groups, focal waves were highlighted over the left and right frontal cortex and a large negative wave was observed over the parietal and occipital cortices (Fig 6, right) (the topography of ERPs for the “different” picture condition are illustrated in S4 Fig.)

During the rotation-related negativity interval (963–1183 ms) (“same” picture condition), there was neither an interaction between group and electrode (F(61, 1708) = 1.610, p = 0.443, partial η2 = 0.035) nor a group effect (F(1, 28) = 0.729, p = 0.400, partial η2 = 0.025). As expected, there was an effect of the variable electrode during the rotation-related negativity interval (F(61, 1708) = 1.505, p = 0.008, partial η2 = 0.051). The electrode effect could be described by a quadratic trend (F(1, 28) = 5.357, p = 0.028). There was neither a linear (F(1, 28) = 0.168, p = 0.685) nor a cubic trend (F(1, 28) = 0.444, p = 0.511) for the electrode effect. The interaction between electrode and group could be described by both a quadratic (F(1, 28) = 0.281, p = 0.045) and a cubic trend (F(1, 28) = 0.258, p = 0.041). No linear trend was observed for the interaction between electrode and group (F(1, 28) = 2.222, p = 0.147).

The statistical results from the topographical analysis during the “same” picture condition showed no interaction between electrode and group (F(1, 28) = 1.236, p = 0.106, partial η2 = 0.038). There was neither a group (F(1, 28) = 0.105, p = 0.748, partial η2 = 0.025) nor an electrode effect for normalized topography (F(1, 28) = 1.035, p = 0.363, partial η2 = 0.044). Also, the electrode effect could not be described by any of linear (F(1, 27) = 0.129, p = 0.722), quadratic (F(1, 27) = 3.781, p = 0.062), or cubic trends (F(1, 27) = 0.166, p = 0.687). The interaction between electrode and group in normalized topography did not showed a linear (F(1, 27) = 2.307, p = 0.140), quadratic (F(1, 27) = 0.434, p = 0.515) or cubic trend (F(1, 27) = 1.540, p = 0.225).

### 3.4 Multiple linear regression and correlations

Multiple linear regression was used to verify whether IQ scores could predict the recorded ERP amplitude values in both experimental groups. For the “same picture” condition, a regression analysis resulted in a statistically significant model for ERP amplitudes recorded in electrode P6 (F (2, 25) = 10.816, p < 0.001, R^2^ = 0.464). ERP values were predicted by both total IQ (β = -0.976, t = -4.216, p < 0.001) and working memory scores (β = 1.043, t = 4.508, p < 0.001). In frontal electrodes, the regression analysis for the “same” picture condition resulted in a statistically significant model for the ERP recorded in electrodes F2 (F (3, 21) = 3.692, p = 0.028, R^2^ = 0.345), F4 (F (2, 25) = 5.834, p = 0.008, R^2^ = 0.318), F5 (F (5, 22) = 6.083, p = 0.001, R^2^ = 0.580), F6 (F (5, 19) = 4.316, p = 0.009, R^2^ = 0.532), F7 (F (2, 25) = 4.076, p = 0.029, R^2^ = 0.246), and F8 (F (2, 22) = 5.366, p = 0.013, R^2^ = 0.328). ERP values were predicted by total IQ (F7: β = 0.797, t = 2.601, p = 0.015; F8: β = 1.025, t = 3.228, p = 0.004), working memory (F2: β = -0.537, t = -2.232, p = 0.037; F4: β = -0.804, t = -3.343, p = 0.003; F5: β = -1.050, t = -3.882, p = 0.001; F6: β = -0.913, t = -2.866, p = 0.010; F7: β = -0.861, t = -2.810, p = 0.009; F8: β = -0.953, t = -3.002, p = 0.007), and verbal comprehension scores (F5: β = 1.394, t = 3.344, p = 0.003; F6: β = 1.094, t = 2.339, p = 0.030) (For the results of multiple linear regression analyses over parietal and frontal electrodes for the “different” picture condition, see S3 Table).

There was no correlation between the ERP amplitudes recorded in parietal electrodes and IQ scores, during the “same” picture condition (Table 7). The ERP amplitude recorded in the electrode F2 (Table 8) showed a moderate negative correlation with processing speed scores (r = -0.498, p = 0.011, Spearman test). We also described a moderate correlation between working memory scores and ERP amplitude in electrodes F4 (r = -0.478, p = 0.010, Spearman test) and F5 (r = -0.437, p = 0.020, Spearman test), whichh disappeared after post hoc correction with adjusted p value < 0.005 (0.05/9).

**Table 7:** Correlations between intelligence scores and parietal ERPs amplitude during “same” pictures condition.

Parietal electrodes—“Same” pictures						
		total IQ	working memory	Processing speed	perceptual organization	verbal comprehension
**P1**	**R**	-0.094	0.068	-0.187	-0.060	-0.058
	**P**	0.627	0.727	0.330	0.758	0.766
**P2**	**R**	0.012	0.161	-0.112	-0.008	0.116
	**P**	0.950	0.413	0.572	0.969	0.557
**P3**	**R**	-0.108	0.135	-0.168	-0.097	-0.104
	**P**	0.584	0.494	0.393	0.624	0.599
**P4**	**R**	-0.076	0.174	-0.004	-0.010	-0.013
	**P**	0.701	0.376	0.983	0.960	0.946
**P5**	**R**	-0.004	0.081	-0.268	0.036	-0.044
	**P**	0.983	0.693	0.185	0.863	0.830
**P6**	**R**	-0.220	0.241	0.019	-0.153	-0.253
	**P**	0.262	0.216	0.923	0.437	0.194
**P7**	**R**	0.128	0.038	-0.045	0.195	0.028
	**P**	0.525	0.851	0.823	0.329	0.889
**P8**	**R**	-0.099	0.169	-0.062	-0.046	-0.207
	**P**	0.623	0.399	0.758	0.822	0.299
**Pz**	**R**	-0.051	0.158	-0.046	-0.020	-0.018
	**P**	0.791	0.414	0.813	0.916	0.925

* statistically significant

**Table 8:** Correlations between intelligence scores and frontal ERPs amplitude during “same” pictures condition.

Frontal electrodes—“Same” pictures						
		total IQ	working memory	processing speed	perceptual organization	verbal comprehension
**F1**	**R**	0.003	-0.314	-0.310	0.078	-0.030
	**P**	0.989	0.111	0.116	0.698	0.881
**F2**	**R**	-0.007	-0.334	-0.498	0.155	-0.133
	**P**	0.974	0.102	0.011*	0.460	0.527
**F3**	**R**	-0.102	-0.317	-0.186	-0.220	-0.013
	**P**	0.597	0.094	0.334	0.253	0.946
**F4**	**R**	-0.072	-0.478	-0.342	-0.349	-0.124
	**P**	0.715	0.010*	0.075	0.069	0.530
**F5**	**R**	-0.148	0.437	-0.111	-0.233	-0.045
	**P**	0.452	0.020*	0.574	0.232	0.821
**F6**	**R**	-0.036	-0.341	-0.037	-0.097	0.067
	**p**	0.863	0.096	0.859	0.646	0.750
**F7**	**R**	0.064	-0.166	0.020	0.035	0.037
	**p**	0.745	0.399	0.920	0.859	0.851
**F8**	**R**	0.229	-0.137	0.084	0.192	0.252
	**p**	0.270	0.515	0.691	0.359	0.224
**Fz**	**R**	0.041	-0.290	-0.334	0.112	0.045
	**p**	0.832	0.127	0.077	0.564	0.818

* statistically significant

## IV. Discussion

We showed that visuospatial ability in intellectually gifted adolescents, measured during a Shepard-Metzler mental rotation task [33], is not characterized by the patterns of parietal cortical activity. Our findings that performance was affected differently by picture condition provides additional support to the hypothesis that non-mirrored (same pictures) and mirrored (different picture) pictures undergo different strategies of processing, as suggested by Hamm and colleagues (2004) [57] and Hung and Hamm (2010) [58].

More specifically, we observed that during the “same” pictures condition, (1) the EEG topographic maps of average intelligence and gifted adolescents were not different during intervals associated with the rotation-related negativity; (2) there was no interhemispheric difference in the amplitude of parietal ERPs of both the gifted and the control groups; (3) interhemispheric differences of ERPs amplitude were observed in frontal ROIs; (4) in both groups, the amplitude of ERPs was larger over the right frontal than over the left hemisphere; and (5) the amplitude of ERPs over the right parietal lobe was predicted by intelligence quotient and working memory scores.

### 4.1 Behavioral results

The main behavioral difference between the experimental groups during the “same” picture condition occurred in task accuracy: the rate of correct responses decreased with the increasing angle of disparity, following mainly a negative linear trend. Also, intellectually gifted adolescents had more correct responses than controls, especially notable for higher angles of disparity.

There was a tendency for slower RTs with an increasing angle of disparity, as shown in Shepard and Metzler’s original study [33], but the average RT of both experimental groups was similar. An fMRI study of math-gifted and average intelligence adolescents reported a similar result [30].

### 4.2 Event-related potentials

According to our results of standard and normalized topography for “same” pictures, average intelligence and gifted adolescents engaged the same cortical regions during the performance of the Shepard-Metzler task, and amplitude of ERPs did not differ between groups.

We did not observe an interhemispheric difference in ERPs amplitude of parietal ROIs, although the activation of the right parietal hemisphere could be predicted by both intelligence quotient and working memory scores. Several studies have presented conflicting results about the laterality of activation of the parietal cortex activation during mental rotation tasks. For instance, lesions of either the right [59] or the left parietal hemispheres [60] are reported to promote impairments in mental rotation performance. Moreover, an absence of interhemispheric differences over the parietal cortex during the Shephard-Metzler task was previously described in an fMRI study [61]. In that study, both the response time and the bilateral activation of the intraparietal sulcus increased with the angular disparity between stimuli. The same results were also described in other fMRI studies [61–64] and were observed in mental rotation tasks using another type of stimuli, such as alphanumeric characters and abstract pictures [62].

Milivojevic and colleagues (2009) suggest that the lateralization effect observed in mental rotation studies using ERP is primarily related to the timing of rotation-related negativity, rather than the extent of cortical involvement [65]. In both hemispheres, the rotation-related negativity begins around 400 ms after stimulus onset, though it lasts a little longer in the left hemisphere (about 60 ms) [65]. Such a difference would not be observed with neuroimaging methods, which have a poorer temporal resolution than EEG [65]. However, Sack and Schuhmann (2012) propose that there are hemispheric differences in activation within the frontoparietal network during spatial imagery [66]. According to these authors, spatial imagery is a multifaceted cognitive construct associated with the segregation of distinct mental processes performed by distinct elements within the frontoparietal network of both hemispheres [66]. For instance, the parietal cortex in each hemisphere is tasked with different processes during spatial imagery, such as mental image generation and spatial analysis [66]. Accordingly, a study using a visuospatial imagery test, the mental clock task, has indicated that the activation of the left parietal cortex occurs before the right side and that the duration of this activation is correlated with RT [67].

### 4.3 Frontoparietal network

As reviewed by Sack and Schμuhmann (2012) [66], the asymmetry of parietal cortex activation may not be the only factor contributing to performance during the Shepard-Metzler mental rotation task. Our results highlighted the asymmetric activation of the frontal cortex during the “same” picture condition in both average intelligence and gifted participants. In both groups, the ERP amplitude over the right frontal hemisphere was larger than the left and could be predicted by total IQ, working memory and verbal comprehension. The data suggest that the difference in accuracy between the experimental groups could be influenced by the frontal cortex functioning. The association between accuracy in mental rotation and fluid intelligence scores had already been previously described in the literature [68].

A previous study about sex differences during the Shepard-Metzler task showed the asymmetric involvement of the frontal cortex during this task [69]. Men had higher accuracy than women when performing the task and presented increased ERP amplitude over the right frontal cortex during the interval of 400–700 ms post-stimulus onset, before the mental rotation itself. In both men and women, the ERP amplitude over the right frontal cortex, before the mental rotation interval, became more negative with increasing angle of disparity [69], which did not occur on the left side.

In summary, our results suggest that a functional interaction between frontal and parietal cortex regions underlie the neural mechanisms associated with intellectual giftedness and the performance of gifted individuals in mental rotation task. Several studies suggest that intelligence is associated with processing efficiency in the integration of information exchanged among many brain regions [70], especially in the frontoparietal network [71, 72]. The interaction between parietal and frontal cortices is implicated in the test of various solutions to a specific problem [26] and has been also associated to others cognitive processes, such as sensory-motor integration [73], spatial working memory [74], visuospatial attention [75], and fluid intelligence [76].

## Supporting information

S1 Fig Aligned blinks and saccades before and after ICA. (TIF)

S2 Fig Accuracy and RT comparison between groups. Accuracy (A) and RT (B) for “different” pictures condition by angle disparity.
(TIF)

S3 Fig Grand-averaged ERPs by angle disparity over the electrode Pz. ERPs of the electrode Pz during the “different” pictures condition of control (A) and gifted adolescents (B). The ERP amplitude over the electrode Pz for “same” pictures (2.106 ± 0.733 μV) was larger than for “different” pictures (1.800 ± 0.736 μV). The average ERPs of each angle of rotation (0°, 50°, 100° and 150°) is color-coded. Values of rotation-related negativity indicate the average of the absolute amplitude of ERPs’ of each group.
(TIF)

S4 Fig Scalp topographies of ERP amplitudes for “different” pictures condition. EEG scalp topographies observed during the rotation-related negativity from 963–1183 ms (for “different” pictures) of control (left) and gifted group (medium), and the difference of ERP amplitude between gifted and control groups (right). Statistical results of standard topography: interaction between group and electrode (F(61, 1708) = 1.016, p = 0.443), group effect (F(1, 28) = 0.729, p = 0.400) and electrode effect (F(61, 1708) = 1.505, p = 0.008). For the electrode effect, there was a quadratic trend (F(1, 27) = 5.357, p = 0.028). Values are color-coded according to absolute ERP amplitude.
(TIF)

S1 Table Behavioral results for “different” pictures condition. (TIF)

S2 Table Multiple linear regression of Accuracy and RT for “different” pictures condition. (TIF)

S3 Table Multiple linear regression of ERPs over parietal and frontal electrodes for “different” pictures condition. (TIF)

## References

[1] Lohman DF. Spatial ability In: Sternberg RJ, editors. Encyclopedia of intelligence. New York: Macmillan; 1994 pp. 1000–1007.

[2] Ferguson ES. The Mind’ s Eye: Nonverbal Thought in Technology: “Thinking with pictures” is an essential strand in the intellectual history of technological development. Science. 1977;197(4306): 827–836. 17730157 10.1126/science.197.4306.827

[3] Lubinski D, Benbow CP. Study of mathematically precocious youth after 35 years: Uncovering antecedents for the development of math-science expertise. Perspect psychol sci. 2006;1(4): 316–345. 10.1111/j.1745-6916.2006.00019.x 26151798

[4] Wai J, Lubinski D, Benbow CP. Spatial ability for STEM domains: Aligning over 50 years of cumulative psychological knowledge solidifies its importance. J Educ Psychol. 2009;101(4): 817–835.

[5] Newcombe NS, Shipley TF. Seeing relationships: Using spatial thinking to teach science, mathematics, and social studies. Am Educ. 2013;37(1): 26.

[6] Andersen L. Visual-Spatial Ability: Important in STEM, Ignored in Gifted Education. Roeper Rev. 2016;36(2): 114–121.

[7] Mathewson JH. Visual‐spatial thinking: An aspect of science overlooked by educators. Sci Educ. 1999;83(1): 33–54.

[8] Pfeiffer SI. Current perspectives on the identification and assessment of gifted students. J Psychoeduc Assess. 2012;30(1): 3–9.

[9] Sękowski AE, Łubianka B. Education of gifted students in Europe. Gift Educ Int. 2015;31(1): 73–90.

[10] Alencar EMLS, Fleith DS, Arancibia V. Gifted education and research on giftedness in South America In: International handbook on giftedness. Dordrecht: Springer; 2009 pp. 1491–1506.

[11] Roid GH. Stanford-Binet Intelligence Scales. 5th ed Itasca: Riverside Publishing; 2003.

[12] Ziegler A, Phillipson SN. Towards a systemic theory of gifted education. High Abil Stud. 2012;23(1): 3–30.

[13] Subotnik RF. A developmental view of giftedness: from being to doing. Roeper Rev. 2003;26(1): 14–15.

[14] Keating DP. Developmental science and giftedness: an integrated life-span framework In: Horowitz FD, Subotnik RF, Matthews DJ, editors. The development of giftedness and talent across the life span. Washington, American Psychological Association; 2009 pp. 189–208.

[15] Kim M. A meta-analysis of the effects of enrichment programs on gifted students. Gift Child Q. 2016;60(2): 102–116.

[16] Gottfredson LS. Mainstream science on intelligence: An editorial with 52 signatories, history, and bibliography. Intelligence. 1997;24(1): 13–23.

[17] McGrew KS. CHC theory and the human cognitive abilities project: Standing on the shoulders of the giants of psychometric intelligence research. Intelligence. 2009;37(1): 1–10.

[18] Carroll JB. Human cognitive abilities: A survey of factor analytic studies. New York: Cambridge University; 1993.

[19] Spearman C. “General Intelligence,” Objectively Determined and Measured. Am J Psychol. 1904;15(2): 201–292.

[20] Hegarty M, Waller D. A dissociation between mental rotation and perspective-taking spatial abilities. Intelligence. 2004;32(2): 175–191.

[21] Gustafsson JE. Schooling and intelligence: effects of track of study on level and profile of cognitive abilities. Int Educ J. 2001;2(4): 166–186.

[22] Pfleiderer B, Ohrmann P, Suslow T, Wolgast M, Gerlach AL, Heindel W, et al N-acetylaspartate levels of left frontal cortex are associated with verbal intelligence in women but not in men: a proton magnetic resonance spectroscopy study. Neuroscience. 2004;123(4): 1053–1058. 10.1016/j.neuroscience.2003.11.008 14751296

[23] Jung RE, Brooks WM, Yeo RA, Chiulli SJ, Weers DC, Sibbitt WL. Biochemical markers of intelligence: a proton MR spectroscopy study of normal human brain. Proc Biol Sci. 1999;266(1426): 1375–1379. 10.1098/rspb.1999.0790 10445292 PMC1690078

[24] Hilger K, Ekman M, Fiebach CJ, Basten U. Intelligence is associated with the modular structure of intrinsic brain networks. Sci Rep. 2017;7(1): 1–12. 29167455 10.1038/s41598-017-15795-7 PMC5700184

[25] Ptak R. The frontoparietal attention network of the human brain: action, saliency, and a priority map of the environment. Neuroscientist. 2012;18(5): 502–515. 10.1177/1073858411409051 21636849

[26] Jung RE, Haier RJ. The Parieto-Frontal Integration Theory (P-FIT) of intelligence: converging neuroimaging evidence. Behav Brain Sci. 2007;30(2): 135–154. 10.1017/S0140525X07001185 17655784

[27] Wendelken C, Ferrer E, Ghetti S, Bailey S, Cutting L, Bunge SA. Fronto-parietal structural connectivity in childhood predicts development of functional connectivity and reasoning ability: a large-scale longitudinal investigation. J Neurosci. 2017;37(35): 8549–8558. 10.1523/JNEUROSCI.3726-16.2017 28821657 PMC5577859

[28] Harris IM, Egan GF, Sonkkila C, Tochon-Danguy HJ, Paxinos G, Watson JD. Selective right parietal lobe activation during mental rotation: a parametric PET study. Brain. 2000;123(1): 65–73. 10611121 10.1093/brain/123.1.65

[29] Just MA, Carpenter PA, Maguire M, Diwadkar V, McMains S. Mental rotation of objects retrieved from memory: A functional MRI study of spatial processing. J Exp Psychol. 2001;130(3): 493–504. 10.1037//0096-3445.130.3.493 11561923

[30] O’Boyle MW, Cunnington R, Silk TJ, Vaughan D, Jackson G, Syngeniotis A, et al Mathematically gifted male adolescents activate a unique brain network during mental rotation. Cogn Brain Res. 2005:25(2): 583–587. 10.1016/j.cogbrainres.2005.08.004 16150579

[31] Coull JT, Frith CD. Differential activation of right superior parietal cortex and intraparietal sulcus by spatial and nonspatial attention. Neuroimage. 1998;8(2): 176–187. 10.1006/nimg.1998.0354 9740760

[32] Desco M, Navas-Sanchez FJ, Sanchez-González J, Reig S, Robles O, Franco C, et al Mathematically gifted adolescents use more extensive and more bilateral areas of the fronto-parietal network than controls during executive functioning and fluid reasoning tasks. Neuroimage. 2011;57(1): 281–292. 10.1016/j.neuroimage.2011.03.063 21463696

[33] Shepard RN, Metzler J. Mental Rotation of Three-Dimensional Objects Abstract. The time required to recognize that two perspective drawings portray. Science. 1971;171(3972): 701–703. 5540314 10.1126/science.171.3972.701

[34] Casey MB, Nuttall R, Benbow CP. The influence of spatial ability on gender differences in mathematics college entrance test-scores across diverse samples. Dev Psychol. 1995;31(4): 697–705

[35] O’Boyle MW, Benbow CP, Alexander JE. Sex differences, hemispheric laterality, and associated brain activity in the intellectually gifted. Dev Neuropsychol. 1995;11(4): 415–443.

[36] Spelke ES. Sex differences in intrinsic aptitude for mathematics and science? A critical review. Am Psychol. 2005;60(9): 950–958. 10.1037/0003-066X.60.9.950 16366817

[37] Borst G, Kievit RA, Thompson WL, Kosslyn SM. Mental rotation is not easily cognitively penetrable. J Cogn Psychol. 2011;23(1): 60–75.

[38] Grabner RH, Ansari D, Reishofer G, Stern E, Ebner F, Neuper C. Individual differences in mathematical competence predict parietal brain activation during mental calculation. Neuroimage. 2007;38(2): 346–356. 10.1016/j.neuroimage.2007.07.041 17851092

[39] Arsalidou M, Taylor MJ. Is 2+ 2 = 4? Meta-analyses of brain areas needed for numbers and calculations. Neuroimage. 2011;54(3): 2382–2393. 10.1016/j.neuroimage.2010.10.009 20946958

[40] Kaufmann L, Wood G, Rubinsten O, Henik A. Meta-analyses of developmental fMRI studies investigating typical and atypical trajectories of number processing and calculation. Dev neuropsychol. 2011;36(6): 763–787. 10.1080/87565641.2010.549884 21761997

[41] Amalric M, Dehaene S. Origins of the brain networks for advanced mathematics in expert mathematicians. Proc Natl Acad Sci. 2016;113(18): 4909–4917. 10.1073/pnas.1603205113 27071124 PMC4983814

[42] Skagerlund K, Bolt T, Nomi JS, Skagenholt M, Västfjäll D, Träff U, et al Disentangling Mathematics from Executive Functions by Investigating Unique Functional Connectivity Patterns Predictive of Mathematics Ability. J Cogn Neurosci. 2018; 1–14. 10.1162/jocn_a_01367 30566368

[43] Hillyard SA, Kutas M. Electrophysiology of cognitive processing. Annu rev Psychol. 1983;34: 33–61. 10.1146/annurev.ps.34.020183.000341 6338812

[44] Rugg MD, Coles MGH. The ERP and cognitive psychology: conceptual issues In: Rugg MD, Coles MGH, editors. Electrophysiology of mind. Oxford: Oxford University Press; 1995 pp. 27–39.

[45] Zacks JM. Neuroimaging studies of mental rotation: A meta-analysis and review. J Cogn Neurosci. 2008;20(1): 1–19. 10.1162/jocn.2008.20013 17919082

[46] Wijers AA, Otten LJ, Feenstra S, Mulder G, Mulder LJ. Brain potentials during selective attention, memory search, and mental rotation. Psychophysiology. 1989; 26(4): 452–467. 10.1111/j.1469-8986.1989.tb01951.x 2798695

[47] Heil M. The functional significance of ERP effects during mental rotation. Psychophysiology. 2002;39(5): 535–545. doi: 10.1017.S0048577202020449 12236320 10.1017/S0048577202020449

[48] Quan C, Li C, Xue J, Yue J, Zhang C. Mirror-normal difference in the late phase of mental rotation: an ERP study. PLoS One. 2017; 12(9): 1–17. 10.1371/journal.pone.0184963 PMC5600392 28915254

[49] Schendan HE, Lucia LC. Visual object cognition precedes but also temporally overlaps mental rotation. Brain Res. 2009;1294: 91–105. 10.1016/j.brainres.2009.07.036 19631629

[50] Milivojevic B, Hamm JP, Corballis MC. Hemispheric dominance for mental rotation: it is a matter of time. Neuroreport. 2009;20(17): 1507–1512. 10.1097/WNR.0b013e32832ea6fd 19829165

[51] Makel MC, Kell HJ, Lubinski D, Putallaz M, Benbow CP. When lightning strikes twice: Profoundly gifted, profoundly accomplished. Psychol Science. 2016;27(7): 1004–1018. 10.1177/0956797616644735 27225220

[52] Wechsler D. Wechsler Intelligence Scale for Children 4 ed Technical and interpretive manual. San Antonio: The Psychological Corporation; 2003.

[53] Wechsler D. Wechsler Adult Intelligence Scale 4 ed Technical manual. San Antonio: The Psychological Corporation; 2008.

[54] Vaivre-Douret L. Developmental and cognitive characteristics of “high level potentialities” (high gifted) children. Int J Pediatr. 2011;2011: 1–14. 10.1155/2011/420297 PMC3184407 21977044

[55] Neubauer AC, Bergner S, Schatz M. Two- vs. three-dimensional presentation of mental rotation tasks: Sex differences and effects of training on performance and brain activation. Intelligence. 2010;38(5): 529–539. 10.1016/j.intell.2010.06.001 20953415 PMC2940390

[56] Ganis G, Kievit R. A New Set of Three-Dimensional Shapes for Investigating Mental Rotation Processes: Validation Data and Stimulus Set. J Open Psychol Data. 2015;3(1): 1–7.

[57] Hamm J, Johnson BW, Corballis M. One good turn deserves another: an event-related brain potential study of rotated mirror–normal letter discriminations. Neuropsychologia. 2004;42(6): 810–820. 10.1016/j.neuropsychologia.2003.11.009 15037059

[58] Kung E, Hamm JP. A model of rotated mirror/normal letter discriminations. Mem Cogn. 2010;38(2): 206–220. 10.3758/MC.38.2.206 20173193

[59] Ditunno PL, Mann VA. Right hemisphere specialization for mental rotation in normals and brain damaged subjects. Cortex. 1990;26(2): 177–188. 10.1016/s0010-9452(13)80349-8 2387155

[60] Mehta Z, Newcombe F. A Role for the Left Hemisphere in Spatial Processing. Cortex. 1991;27(2): 153–167. 10.1016/s0010-9452(13)80121-9 1879146

[61] Carpenter PA, Just MA, Keller TA, Eddy W, Thulborn K. Graded functional activation in the visuospatial system with the amount of task demand. J Cog Neurosci. 1999;11(1): 9–24. 10.1162/089892999563210 9950711

[62] Jordan K, Heinze HJ, Lutz K, Kanowski M, Jäncke L. Cortical activations during the mental rotation of different visual objects. Neuroimage. 2001;13(1): 143–152. 10.1006/nimg.2000.0677 11133317

[63] Cohen MS, Kosslyn SM, Breiter HC, DiGirolamo GJ, Thompson WL, Anderson K, et al Changes in cortical activity during mental rotation. A mapping study using functional MRI. Brain. 1996;119(1): 89–100. 8624697 10.1093/brain/119.1.89

[64] Kosslyn SM, DiGirolamo GJ, Thompson WL, Alpert NM. Mental rotation of objects versus hands: Neural mechanisms revealed by positron emission tomography. Psychophysiology. 1998;35(2): 151–161. 9529941

[65] Milivojevic B, Hamm JP, Corballis MC. Hemispheric dominance for mental rotation: it is a matter of time. Neuroreport. 2009;20(17): 1507–1512. 10.1097/WNR.0b013e32832ea6fd 19829165

[66] Sack AT, Schuhmann T. Hemispheric differences within the fronto-parietal network dynamics underlying spatial imagery. Front Psychol. 2012;3: 1–10. 22754546 10.3389/fpsyg.2012.00214 PMC3385155

[67] Formisano E, Linden DEJ, Salle F, Trojano L, Esposito F, Sack AT, et al Tracking the Mind’ s Image in the Brain I: Time-Resolved fMRI during Visuospatial Mental Imagery. Neuron. 2002;35(1): 185–194. 10.1016/s0896-6273(02)00747-x 12123618

[68] Varriale V, van der Molen MW, De Pascalis V. Mental rotation and fluid intelligence: A brain potential analysis. J Intell. 2018;69: 146–157.

[69] Yu Q, Tang Y, Li J, Lu Q, Wang H, Sui D, et al Sex differences of event-related potential effects during three-dimensional mental rotation. NeuroReport. 2009;20(1): 43–47. 10.1097/WNR.0b013e32831c50f4 19057281

[70] Heuvel MP, Stam CJ, Kahn RS, Hulshoff HE. Efficiency of Functional Brain Networks and Intellectual Performance. J Neurosci. 2009;29(23): 7619–7624. 10.1523/JNEUROSCI.1443-09.2009 19515930 PMC6665421

[71] Cole MW, Bagic A, Kass R, Schneider W. Prefrontal Dynamics Underlying Rapid Instructed Task Learning Reverse with Practice. J Neurosci. 2010;30(42): 14245–14254. 10.1523/JNEUROSCI.1662-10.2010 20962245 PMC3128837

[72] Cole MW, Yarkoni T, Repovs G, Anticevic A, Braver TS. Global Connectivity of Prefrontal Cortex Predicts Cognitive Control and Intelligence. J Neurosci. 2012;32(26): 8988–8999. 10.1523/JNEUROSCI.0536-12.2012 22745498 PMC3392686

[73] Rizzolatti G, Luppino G, Matelli M. The organization of the cortical motor system: New concepts. Electroencephalogr Clin Neurophysiol. 1998;106(4): 283–296. 10.1016/s0013-4694(98)00022-4 9741757

[74] Rottschy C, Langner R, Dogan I, Reetz K, Laird AR, Schulz JB, et al Modelling neural correlates of working memory: A coordinate-based meta-analysis. Neuroimage. 2012;60(1): 830–846. 10.1016/j.neuroimage.2011.11.050 22178808 PMC3288533

[75] Corbetta M, Shulman G. Spatial neglect and attention networks. Annu Rev Neurosci. 2011;(34): 569–599. 21692662 10.1146/annurev-neuro-061010-113731 PMC3790661

[76] Preusse F, Elke M, Deshpande G, Krueger F, Wartenburger I. Fluid Intelligence Allows Flexible Recruitment of the Parieto-Frontal Network in Analogical Reasoning. Front Hum Neurosci. 2011;5(22): 1–14. 21415916 10.3389/fnhum.2011.00022 PMC3049247

