# Supplementary figures and images for: The role of frontal and parietal cortex in the performance of gifted and average adolescents in a mental rotation task
Source: PLoS One. 2020 May 13;15(5):e0232660. doi: 10.1371/journal.pone.0232660 (PMC7219753; doi:10.1371/journal.pone.0232660)

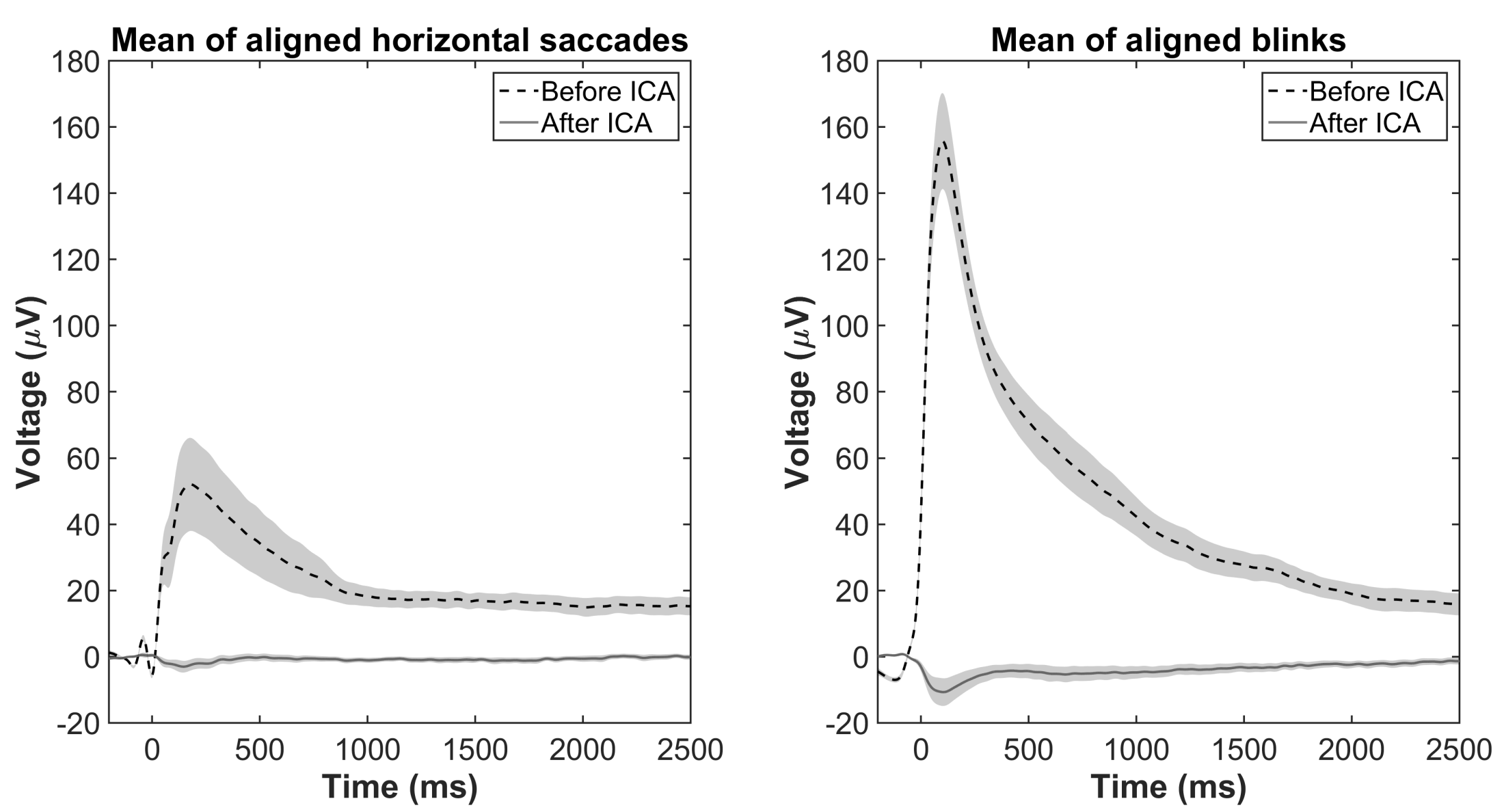

Supplement: S1 Fig — (TIF) [file pone.0232660.s001.tif]

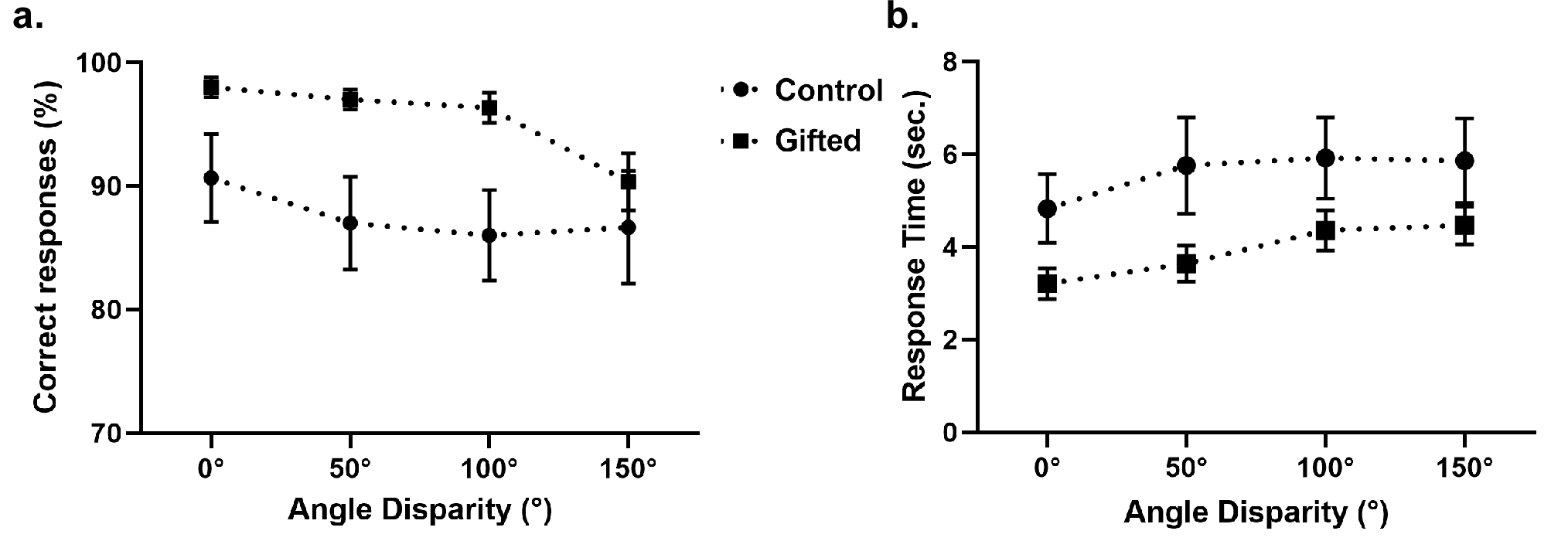

Supplement: S2 Fig — Accuracy (A) and RT (B) for “different” pictures condition by angle disparity. (TIF) [file pone.0232660.s002.tif]

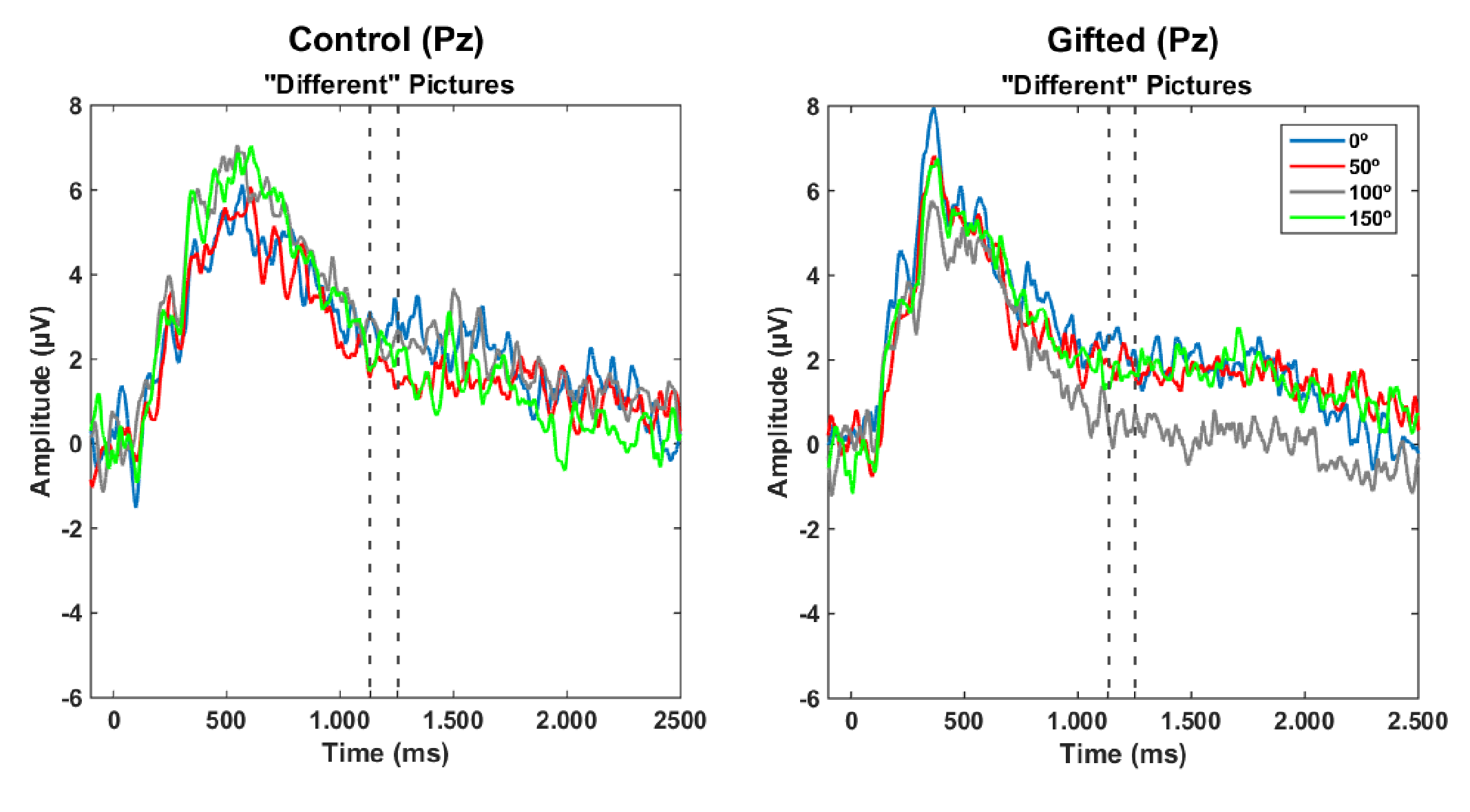

Supplement: S3 Fig — ERPs of the electrode Pz during the “different” pictures condition of control (A) and gifted adolescents (B). The ERP amplitude over the electrode Pz for “same” pictures (2.106 ± 0.733 μV) was larger than for “different” pictures (1.800 ± 0.736 μV). The average ERPs of each angle of rotation (0°, 50°, 100° and 150°) is color-coded. Values of rotation-related negativity indicate the average of the absolute amplitude of ERPs’ of each group. (TIF) [file pone.0232660.s003.tif]

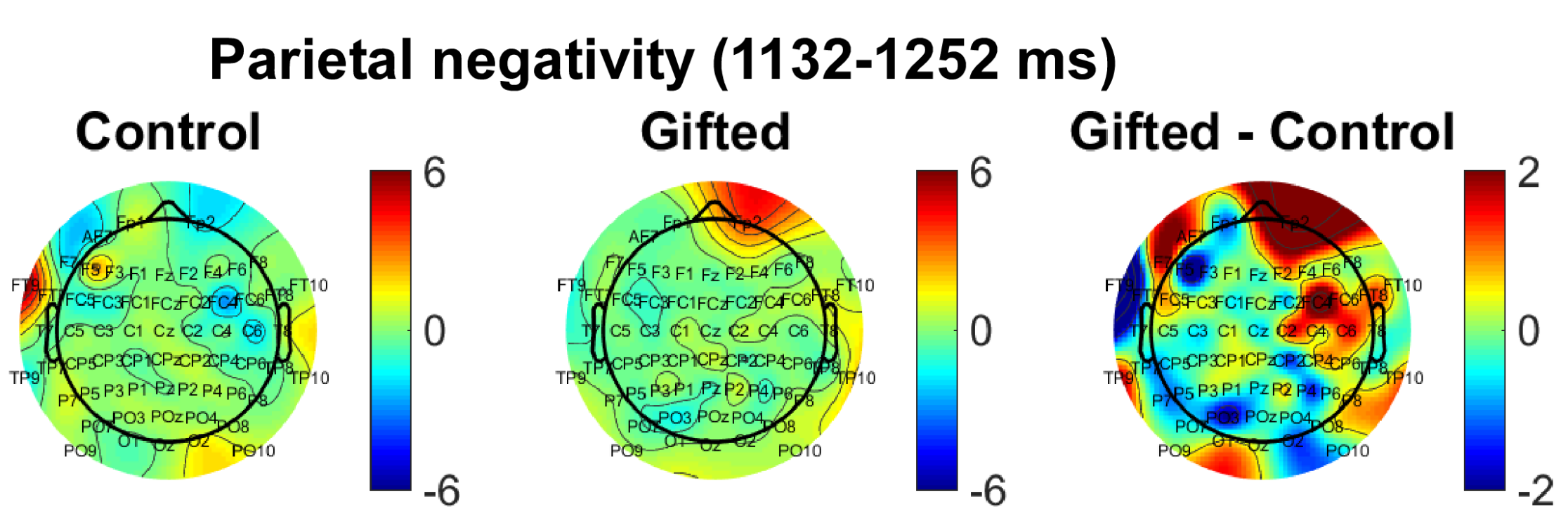

Supplement: S4 Fig — EEG scalp topographies observed during the rotation-related negativity from 963–1183 ms (for “different” pictures) of control (left) and gifted group (medium), and the difference of ERP amplitude between gifted and control groups (right). Statistical results of standard topography: interaction between group and electrode (F(61, 1708) = 1.016, p = 0.443), group effect (F(1, 28) = 0.729, p = 0.400) and electrode effect (F(61, 1708) = 1.505, p = 0.008). For the electrode effect, there was a quadratic trend (F(1, 27) = 5.357, p = 0.028). Values are color-coded according to absolute ERP amplitude. (TIF) [file pone.0232660.s004.tif]

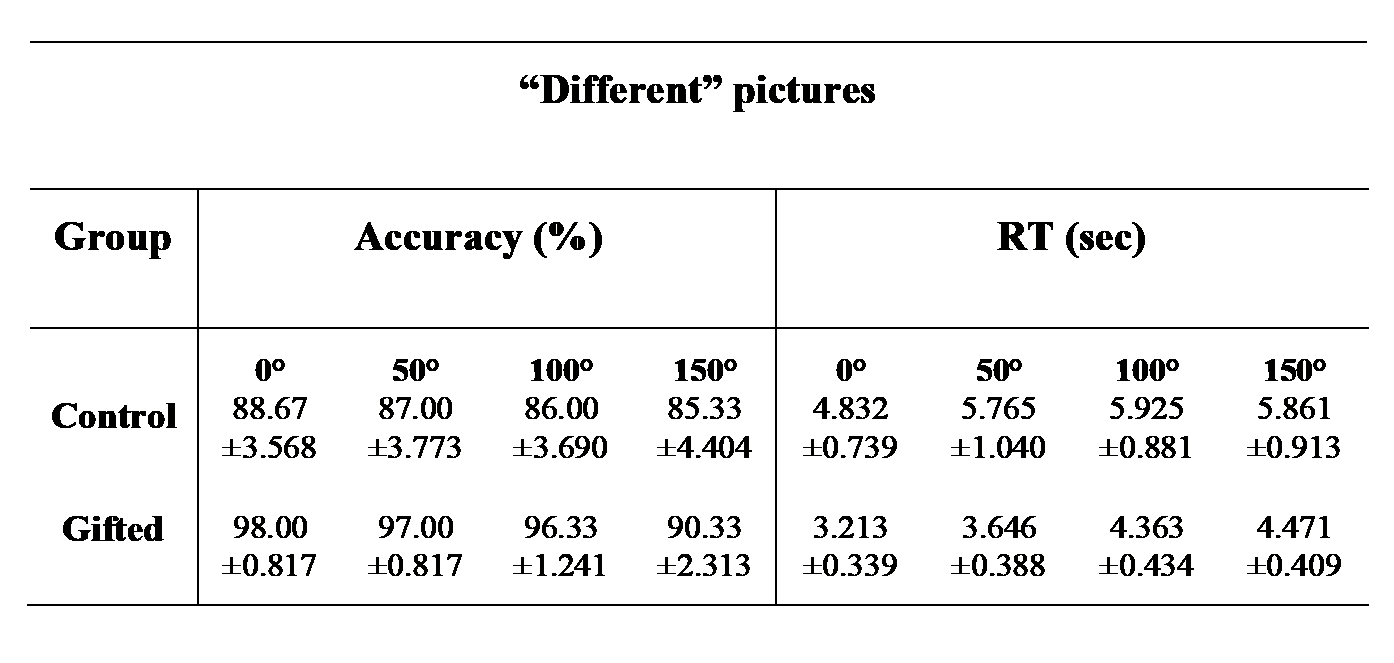

Supplement: S1 Table — (TIF) [file pone.0232660.s005.tif]

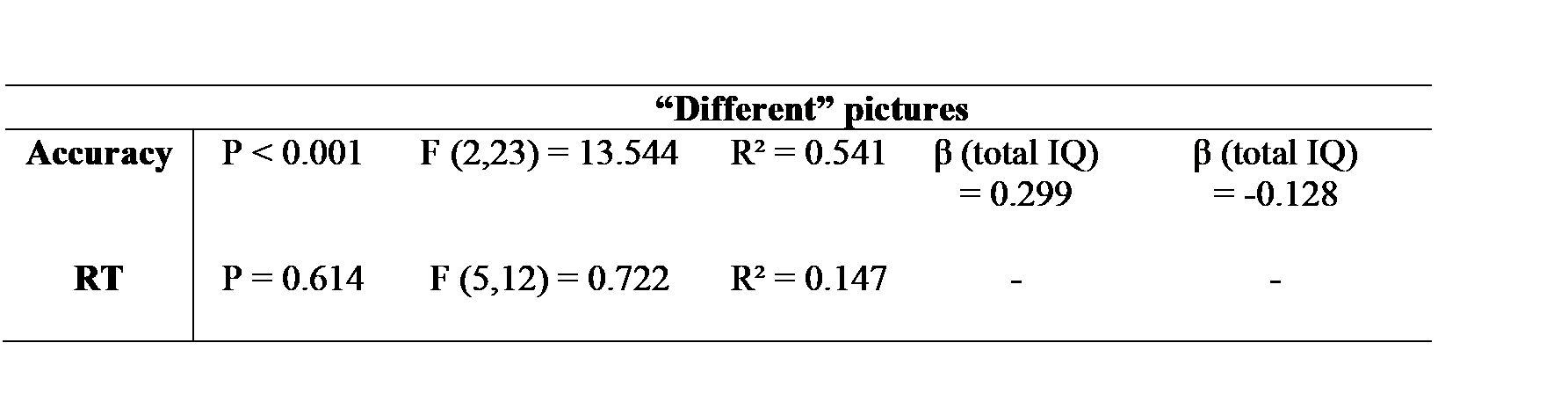

Supplement: S2 Table — (TIF) [file pone.0232660.s006.tif]

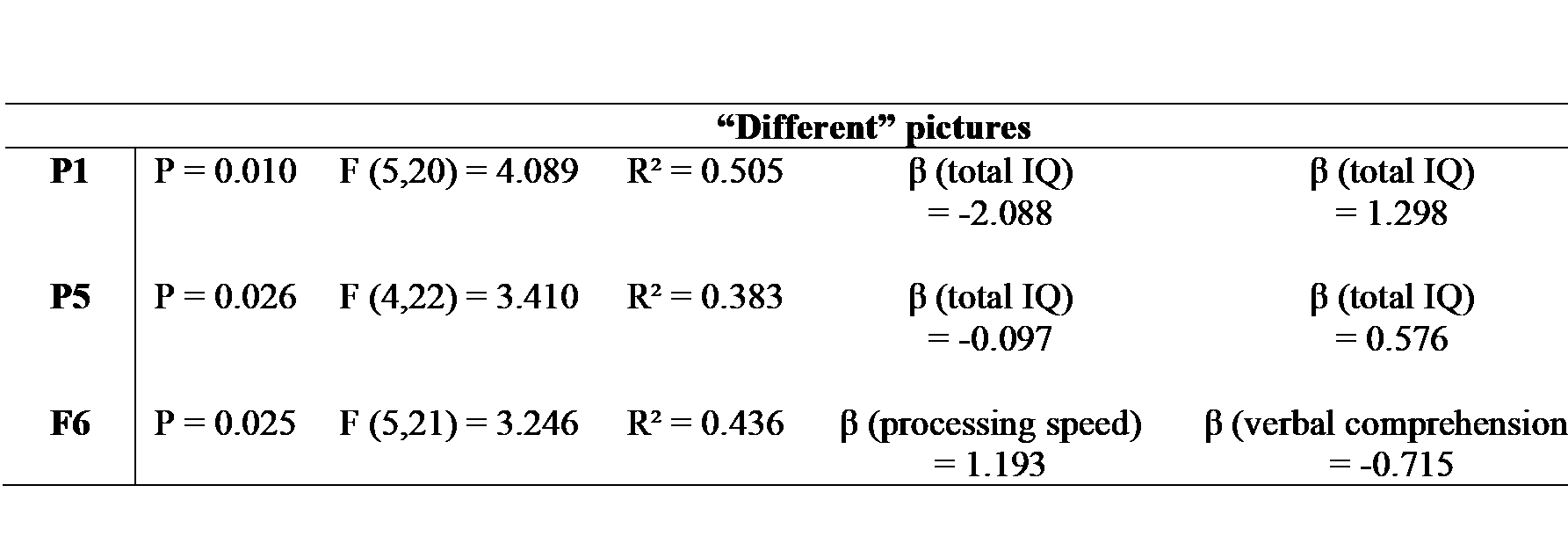

Supplement: S3 Table — (TIF) [file pone.0232660.s007.tif]
